# Biomechanical and Occlusal Factors Influencing the Longevity of Single-Unit Restorations: A Comprehensive Review

**DOI:** 10.7759/cureus.85998

**Published:** 2025-06-14

**Authors:** Wedad S Alaida, Safa A Gadi, Rokia E Al-Ghannam, Moayad F Alamri, Feras I Mirdad, Ruba M Argaibeh, Bushra A Alqahtani, Abdulrahman M Alqahtani, Abdulelah A Al Jaban, Turki M Alkuraydimi, Abdulrahman S Alamari

**Affiliations:** 1 Prosthodontics, Ministry of Health, Riyadh, SAU; 2 Pediatric Dentistry, Ministry of Health, Riyadh, SAU; 3 Dentistry, Ministry of Health, Riyadh, SAU; 4 Family Dentistry, King Abdulaziz University, Jeddah, SAU; 5 Prosthodontics, King Abdulaziz University Hospital, Jeddah, SAU; 6 Dentistry, Mustaqbal University, Buraydah, SAU; 7 Dentistry, Armed Forces Hospital Southern Region, Abha, SAU; 8 General Dentistry, Ministry of Health, Riyadh, SAU; 9 Dentistry, King Abdulaziz Medical City Riyadh, Riyadh, SAU; 10 Dentistry, Umm Al-Qura University, Mecca, SAU; 11 Family Dentistry, Ministry of Health, Riyadh, SAU

**Keywords:** biomechanics in dentistry, crown failure, occlusal forces, prosthodontic complications, single-unit restorations

## Abstract

The long-term success of single-unit restorations is a key objective in restorative dentistry; however, failure rates remain significant due to multiple contributing factors, including inadequate tooth preparation, inappropriate material selection, bonding deficiencies, and occlusal mismanagement. This narrative review aimed to critically assess clinical studies and systematic reviews published between 2000 and 2024 that explored the biomechanical and occlusal determinants affecting the longevity of tooth-supported crowns. A structured search was performed using PubMed, Scopus, and Web of Science databases, supplemented by manual reference screening. Observations from the literature highlight several influential risk factors such as loss of tooth vitality, improper occlusal adjustment, bruxism, and poor margin design. Additionally, finite element analysis studies were reviewed to understand how material properties and preparation geometry influence stress distribution and fatigue. These findings highlight how small, well-considered decisions like refining occlusal contacts, choosing the right material, or adjusting the preparation design can make a meaningful difference in preventing complications. By drawing from the current evidence, this review reinforces the value of thoughtful, evidence-based clinical practices and points to specific areas where further research could help make single-unit restorations more reliable and long-lasting.

## Introduction and background

The decision to replace a single missing tooth is frequently influenced by the poor prognosis of restoring the original tooth. Teeth that are severely compromised due to recurrent caries, trauma, endodontic failure, or periodontal disease often require extraction, followed by prosthetic rehabilitation to restore function and esthetics [1]. Within this restorative framework, occlusion plays a fundamental role. The concept of occlusion has evolved from a focus on static tooth contacts to encompass dynamic, functional, and neuromuscular interactions involving the dentition, masticatory muscles, and temporomandibular joints (TMJs) during various activities such as mastication, speech, and rest [2]. However, despite improvements in materials and techniques, single-unit restorations continue to experience failures due to biomechanical and occlusal factors that are not always well understood or consistently addressed in clinical practice.

To understand these failures, it is essential to consider the role of occlusion, not only as the way teeth meet during biting, but as a dynamic system involving the teeth, jaw muscles, and TMJs. This expanded understanding has roots in the field of gnathology, which comprehensively addresses the biological and mechanical functions of the masticatory system. According to the Glossary of Occlusal Terms by the International Academy of Gnathology (1979), gnathology encompasses morphology, anatomy, physiology, pathology, and therapeutic considerations related to the jaws and teeth, with implications for the entire body [3]. Historical developments in this discipline led to the refinement of occlusal recording techniques and emphasised the importance of mandibular dynamics, centric relation, and occlusal vertical dimension as essential elements in restorative dentistry [4]. Although the emphasis on gnathology in undergraduate dental education declined after the 1970s, its principles remain integral to postgraduate training and advanced clinical practice [5].

Core occlusal principles, such as centric relation, a reproducible and stable mandibular position independent of tooth contact, serve as reliable references even in single-unit restorations, particularly when addressing patients with occlusal disharmony or parafunctional habits [6]. Anterior guidance protects posterior teeth during mandibular excursions, and the maintenance of occlusal vertical dimension supports both functional efficiency and esthetic harmony [6]. Intercuspal design ensures proper force distribution during mastication, while a nuanced understanding of mandibular movements, both rotational and translational, is essential for fabricating restorations that can adapt to functional dynamics [7]. With these principles forming the foundation for sound occlusal planning, the present review aims to explore the biomechanical and occlusal determinants that influence the long-term success of single-unit restorations. By bringing together the latest evidence, this review aims to support clinicians in making more informed choices about occlusal design, material selection, and how forces are managed, ultimately helping to create restorations that last longer and perform better in everyday function.

## Review

Materials and methods

This narrative review employed a structured search strategy to identify and synthesise relevant literature on biomechanical and occlusal factors affecting single-unit restorations. Electronic searches were conducted using three major databases: PubMed, Scopus, and Web of Science, covering the period from January 2000 to April 2024. Keywords were applied both individually and in Boolean combinations, including: “single crown failure,” “occlusal adjustment,” “bruxism,” “restorative material properties,” and “prosthodontic complications.” The search was augmented by the manual examination of reference lists from pertinent sources, including selected review articles and original research papers, to capture additional relevant studies.

The inclusion criteria encompassed clinical trials, observational studies, and in vitro studies that investigated the mechanical performance, failure modes, occlusal considerations, or long-term outcomes of single-unit restorations. Studies were selected if they specifically addressed prosthodontic or restorative implications. Exclusion criteria included articles not published in English, studies lacking accessible full texts, and research unrelated to prosthodontic restorations, such as purely orthodontic topics. Case reports, expert opinions, and editorials were also excluded.

Although this was not a systematic review, careful selection and synthesis procedures were applied to ensure inclusion of high-quality, clinically relevant, and representative literature from the last two decades.

Fundamentals of occlusal forces

Definitions and Terminologies

The following definitions are adapted from the Glossary of Prosthodontic Terms: Tenth Edition (GPT-2023) to ensure standardization and clarity in prosthodontic language [5].

Occlusion: The static relationship between the incising or masticating surfaces of the maxillary or mandibular teeth or tooth analogs.

Occlusal force: The force developed in the masticatory system as a result of muscle activity and function, including both functional and parafunctional contacts.

Axial force: A force directed along the long axis of a tooth or implant; typically considered favorable in stress distribution.

Occlusal overload: Excessive occlusal force that exceeds the adaptive capacity of the periodontium or prosthesis, potentially leading to structural or physiological damage.

Masticatory force: The dynamic force produced by the muscles of mastication during the act of chewing.

Occlusal trauma: An injury to the periodontium resulting from occlusal forces that exceed its capacity to adapt or repair. It may be classified as acute or chronic.

Primary occlusal trauma: Occlusal trauma occurring on a tooth or teeth with normal periodontal support as a result of excessive occlusal forces.

Secondary occlusal trauma: Occlusal trauma occurs when normal occlusal forces are applied to a tooth or teeth with compromised periodontal support.

Types of occlusions

According to McNeill [6], occlusions can be broadly classified into three general types: physiologic occlusions, non-physiologic occlusions, and treatment occlusions [6]. A physiologic occlusion refers to an occlusal scheme that functions without causing discomfort or dysfunction in the masticatory system. It supports normal function and is well adapted to the patient’s anatomy and neuromuscular coordination [6]. In contrast, a non-physiologic occlusion denotes an occlusal relationship that results in pathologic changes or symptoms, such as pain, abnormal tooth wear, or TMJ disorders. This type often requires intervention due to its disruptive impact on oral health and function [6]. Finally, a treatment occlusion is the occlusal scheme established through clinical procedures, such as restorations or orthodontic treatments. The goal of a treatment occlusion is to create a stable and functional occlusal relationship that mimics or restores physiologic conditions, particularly in patients with compromised natural occlusions [6].

Physiologic Occlusion

A physiologic occlusion, also known as a “normal” occlusion, suggests that disease and/or dysfunction are not present and treatment is not required. Essentially, it is defined as an occlusion in which a functional equilibrium or state of homeostasis exists within the tissues of the masticatory system [6]. Throughout life, masticatory tissues adapt to maintain physiological equilibrium by responding to internal biological factors, external environmental factors, and time-dependent changes. This continuous adaptation ensures the integrity and functionality of the masticatory system. In addition, the fibrous connective tissues, as well as the mesenchymal layers of the TMJ, adapt by continual progressive and regressive remodelling [6,7].

Non-physiologic Occlusion

A non-physiologic occlusion is defined as an occlusion in which the tissues of the masticatory system have lost their functional equilibrium or hemostasis in response to the functional demand, injury, or disease [6]. Commonly referred to as a “traumatic” or “pathologic” occlusion, it suggests that limited disease and/or dysfunction is present, and treatment may be required. Pathologic changes can result from sudden or abrupt insults or from loading of sufficient magnitude or duration that results in insufficient opportunity for tissue adaptation. These sudden, disruptive changes can be caused by trauma, including parafunction, inflammation, or disease, and, at times, by iatrogenic causes [7,8].

Treatment Occlusion

A treatment occlusion is often referred to as an “ideal” or “therapeutic” occlusion. It indicates that specific treatment criteria are required to treat the effects of trauma or disease [6].

Impact of occlusal forces on dental structures

Occlusal forces, when excessive or improperly distributed, can adversely affect various components of the oral system. The periodontium is particularly vulnerable, as excessive occlusal loading may result in occlusal trauma characterized by increased tooth mobility, widening of the periodontal ligament space, and eventual alveolar bone resorption [9]. Tooth structure integrity can also be compromised by repetitive occlusal stress. Such forces may lead to enamel wear, dentinal microcracks, and progressive structural weakening of the tooth [10]. Finite element analysis (FEA) has shown that peak cervical stress often concentrates asymmetrically at the cervical enamel surface, particularly in the mesiobuccal line angle area, signifying a potential site for non-carious cervical lesions and structural fatigue [11].

The TMJ is another critical structure affected by occlusal imbalance. Prolonged or excessive occlusal forces may be transmitted to the TMJ, contributing to joint overload, inflammation, and long-term degeneration - all hallmarks of temporomandibular disorders (TMDs) [12]. Additionally, high occlusal stresses are a well-documented cause of restorative failures. These include microfractures, debonding, and material fatigue, all of which can significantly compromise the function and longevity of dental restorations [13]. Understanding the multidimensional impact of occlusal forces is essential for effective diagnosis, treatment planning, and maintenance of both natural and restored dentition.

Biomechanics of single-unit restorations

Stress distribution within single-unit restorations plays a pivotal role in their longevity and clinical performance. The transmission of occlusal forces through a restoration to the underlying tooth structure depends on various biomechanical factors, including the material properties of the restorative material, the geometric design of the restoration, and the quality of the tooth preparation. An optimal biomechanical design aims to evenly distribute functional loads while minimizing stress concentrations that may promote mechanical failure or biological complications [14].

According to Santos et al. [14], the failure of single-unit restorations is a multifactorial phenomenon influenced by several interrelated elements [14]. Tooth-related factors include the presence of multiple-surface restorations, which increase mechanical complexity and the likelihood of failure. Deep cervical margins can compromise the marginal seal, reducing the restoration’s resistance to microleakage and recurrent caries. Additionally, the location of the tooth significantly affects stress distribution, with posterior teeth being subjected to higher occlusal loads. Tooth vitality also influences outcomes, as restorations performed on vital teeth generally demonstrate better survival rates [14].

Patient-related factors also play a significant role. Systemic health conditions, caries risk, and demographic variables such as age and sex can impact restoration success. Parafunctional habits like bruxism and lifestyle choices, including smoking, exert mechanical and biological stresses on restorations. Furthermore, overall periodontal health, the number of existing restorations, and socioeconomic status are associated with long-term restoration outcomes [14].

Dentist-related factors such as clinical expertise, decision-making ability, and manual skills directly influence the quality and durability of the final restoration. Additionally, technique-related factors, including proper moisture isolation and adherence to evidence-based protocols, are essential for clinical success. Even the type of dental practice, such as a large group versus a solo practice, has an influence on restoration outcomes, likely due to differences in available technology, workload distribution, and procedural standardization [14].

It is therefore crucial to incorporate these biomechanical considerations into treatment planning, as they can significantly enhance the functional integrity and durability of single-unit restorations.

FEA studies

FEA is a well-established tool for modelling stress distribution in dental restorations under simulated occlusal loading. Studies using FEA have demonstrated that crown geometry, material stiffness, and margin design significantly influence the transfer of stress to both the restoration and the underlying tooth structure [15-33]. For example, Özkir [16] showed that ceramic crowns exhibited higher internal stress compared to composites, while bulk-fill composites concentrated stress at the adhesive interface. Tribst et al. [17] found that materials with a high elastic modulus reduced stress on the abutment but increased it within the crown itself. Preparation design was also shown to play a key role in force transmission; Abid and Mohammed [20] reported that a palatal chamfer in lithium disilicate veneers resulted in more favourable stress patterns, and Zarone et al. [21] observed that chamfer with palatal overlap most closely mimicked the stress distribution seen in natural teeth. Additional FEA-based outcomes related to restorative material choice, tooth preparation geometry, and occlusal load orientation are discussed in detail in the subsequent sections. Together, these studies highlight the clinical relevance of FEA in optimizing single-unit restoration performance.

Material properties

The choice of restorative material plays a critical role in managing occlusal stress, particularly in endodontically treated teeth. Gurbuz et al. [15] concluded that both the mechanical properties and the elastic modulus of restorative materials significantly influence stress distribution. Materials with differing elastic moduli respond to occlusal forces in distinct ways, which affects how stress is transferred to the underlying tooth structure.

A FEA study conducted by Özkir SE [16] further highlighted the influence of restorative material selection on stress distribution in partial crowns. The study found that ceramic materials produced the highest stress concentration within the restoration itself, while bulk-fill composites exhibited the highest stress levels at the interface of the tooth structure and adhesive system [16]. This finding underscores the complex interplay between restorative materials and the biological substrate.

It has been widely observed that materials with a higher elastic modulus are associated with a greater concentration of stress within the restorative structure [17]. Materials with a high modulus of elasticity, such as zirconia, tend to transmit occlusal forces more rigidly, which may protect the underlying tooth but concentrate stress within the restoration. Conversely, more resilient materials absorb forces differently, distributing stress more evenly but potentially transferring it to other components of the restoration complex [18].

Tribst et al. [17] conducted a study that further clarified this relationship. They concluded that crowns with a high elastic modulus effectively decreased stress levels in the abutments, while crowns composed of materials with a low elastic modulus helped reduce stress within the crown itself [17]. These findings emphasize the need for careful material selection based on the specific clinical needs of the patient and desired biomechanical behavior.

Preparation design

Tooth preparation design is a pivotal factor influencing the distribution of occlusal stress in restorations. Key parameters such as the degree of taper, the amount of tooth reduction, and the configuration of the finish line directly affect the transmission of occlusal forces through the restoration and the underlying tooth structure [18,19]. Güntekin et al. [18] demonstrated that variations in margin design for zirconia crowns significantly alter stress patterns. Additionally, they highlighted that even minor modifications in preparation geometry can lead to notable changes in force distribution [18].

In a 2023 FEA study, Abid and Mohammed [20] reported that a palatal chamfer design for lithium disilicate laminate veneers provided more favorable stress distribution compared to other preparation types [20]. Similarly, an earlier FEA study by Zarone et al. [21] found that in porcelain veneers, the chamfer with palatal overlap preparation more closely replicated the natural stress distribution observed in intact teeth when compared to the window technique, suggesting superior biomechanical performance under load [21].

Tiu et al. [22] conducted a systematic review focusing on clinical tooth preparations and associated measurement methods. Their findings indicated that total occlusal convergence is the most critical preparation parameter contributing to the retention and resistance of cemented complete crowns [22]. Similarly, Abu Hassan et al. observed that bevel and chamfer margin designs exhibited higher stress concentrations under vertical loading conditions when compared to the shoulder margin design, which appeared to distribute forces more effectively [23].

Furthermore, studies by Oberoi and Hegde [24] and McCoy [25] underscore the long-established principle of directing occlusal forces along the long axis of the tooth. This biomechanical alignment is essential for minimizing lateral stresses and reducing the likelihood of failure, particularly in restorations subjected to repetitive masticatory loads.

Influence of occlusal contacts and anatomical factors

The manner in which occlusal contacts are established plays a critical role in the distribution of functional stresses. Ideally, during maximum intercuspation, occlusal forces should be directed along the tooth’s long axis to maintain biomechanical harmony. However, deviations resulting from improper contact points can generate localized areas of high stress concentration and potentially compromise the integrity of the restoration. A study by Kim et al. investigated stress distribution in crowns with varying cusp angles and found that crowns with steeper cuspal inclines exhibited the highest levels of maximum principal stress; the finding suggests that cusp geometry directly influences stress magnitude [26]. Similarly, research examining the geometry of tooth preparation revealed a significant correlation between anatomical preparation and stress reduction. Models with anatomical preparation demonstrated lower stress values compared to non-anatomical configurations, indicating a biomechanical advantage to maintaining natural contours during preparation [27].

Moreover, a positive correlation has been reported between ferrule height and stress distribution at both the abutment-post and abutment-root interfaces. Increased ferrule height led to a more favorable distribution of stresses and contributed to the structural reinforcement of the restored tooth [28]. Borges et al. [29] studied the occlusal anatomy of computer-aided design/computer-aided manufacturing (CAD/CAM)-fabricated feldspathic posterior crowns and its influence on stress concentration and fracture resistance. Their findings revealed that an anatomic design featuring reduced cusp angulation and a less pronounced occlusal sulcus can decrease stress concentrations and improve fracture load capacity, thereby enhancing the mechanical performance of feldspathic CAD/CAM crowns [29].

Fatigue and microcrack propagation

Over time, cyclic loading from mastication can induce fatigue in both the restorative material and the supporting natural tooth structure. An in vitro study by Zhang et al. [30] demonstrated that ceramics are particularly susceptible to fatigue-related degradation. The authors reported a significant reduction, up to a factor of two, in load-bearing capacity and critical bite force after cyclic loading. This is equivalent to one year of functional use, underscoring the long-term impact of fatigue on ceramic restorations.

Nalla et al. [31] investigated the mechanisms of fatigue crack propagation in dentin and emphasized the importance of minimizing stress concentrations to reduce the risk of long-term structural failure. Building on this, Kruzic et al. [32] evaluated the frequency-dependent nature of crack growth in dentin. Their findings suggested that fatigue-related crack progression occurs via a cycle-dependent mechanism involving alternating phases of crack-tip blunting and resharpening. Although no definitive fatigue striations were observed on fracture surfaces, these microstructural changes provided insight into the underlying mechanics of dentin degradation under repetitive loading.

Similarly, Frankenberger et al. [33] conducted an in vitro study comparing post-fatigue fracture resistance and marginal integrity among various restorative approaches in endodontically treated teeth, including partial crowns, full crowns, endocrowns, and fiber-reinforced resin composites. Their results indicated that intra-coronal fiber-reinforced composite restorations exhibited more gap-free margins in enamel compared to conventional resin composites. Additionally, indirect restorations performed significantly better than direct restorations in terms of fracture resistance. This indicates that when proper restorative techniques and materials are used, superior long-term performance can be achieved [33].

Overview of restorative materials

In restorative dentistry, a variety of materials are employed to repair and reconstruct tooth structure. Each material possesses distinct properties and clinical applications.

Dental Amalgam

This is a traditional material composed of a mixture of metals, including silver, mercury, tin, and copper. Known for its durability and strength, amalgam has been widely used for posterior restorations. However, due to concerns about aesthetics and mercury content, its usage has declined in favor of tooth-colored alternatives [34].

Composite Resins

These tooth-colored materials are made from an organic polymer matrix (typically bisphenol A-glycidyl methacrylate (bis-GMA)) reinforced with inorganic fillers like silica. They offer excellent aesthetics and are suitable for both anterior and posterior restorations. Furthermore, advances in nanotechnology have led to the development of nanocomposites, which possess improved mechanical properties and polishability [35].

Glass Ionomer Cements (GICs)

Comprising a silicate glass powder and an aqueous solution of polyacrylic acid, GICs chemically bond to tooth structure and release fluoride, providing anticariogenic benefits. They are commonly used in non-load-bearing areas, such as cervical lesions and as liners or bases [36]. They have not only compressive strengths comparable to those of composite resins but also reasonable flexural strength [37].

Modified forms of glass ionomers are available in the form of resin-modified glass ionomers and glass carbomers. The physical properties of these materials are comparable to those of conventional glass-ionomers, but their biocompatibility is less favorable [37].

Ceramics

Dental ceramics, including porcelain and lithium disilicate, are prized for their superior aesthetics and biocompatibility. They are predominantly used for crowns, veneers, inlays, and onlays. While traditional feldspathic porcelains offer excellent translucency, newer materials such as zirconia provide enhanced strength suitable for posterior restorations [38].

Gold Alloys

Gold restorations are traditionally esteemed for their exceptional durability, biocompatibility, and wear properties. Despite their longevity, their metallic appearance and cost have limited their acceptance among patients seeking aesthetic solutions [39].

Zirconia

Zirconia is a high-strength ceramic that has revolutionized restorative dentistry with its excellent mechanical properties. It exhibits high fracture toughness [40] and compressive strength values of 2,000 MPa. It also has an elastic modulus of approximately 3,895 MPa, which promotes durable restorations [41]. Zirconia’s transformation-toughening mechanism provides resistance against crack propagation, making it particularly suitable for posterior crowns and implant-supported restorations. The advent of CAD/CAM technology has further enhanced the precision and clinical outcomes of zirconia restorations [42].

Polyether Ether Ketone (PEEK)

PEEK is a high-performance thermoplastic polymer increasingly utilized in dentistry for its favorable biomechanical characteristics. With an elastic modulus closer to that of cortical bone (approximately 3-4 GPa), PEEK offers excellent shock absorption and stress distribution properties, reducing the risk of stress shielding in implant-supported prostheses [43]. Its biocompatibility, chemical resistance, and radiolucency render it an attractive alternative for frameworks and substructures. However, its relatively lower aesthetic appeal and evolving clinical data require careful consideration when used in highly visible regions [44].

Comparative analysis of material properties

Mechanical Strength and Elastic Modulus

Due to their exceptional mechanical properties, ceramics such as zirconia are widely used in high-stress dental applications. Zirconia exhibits a high compressive strength of approximately 2,000 MPa and an elastic modulus of around 3,895 MPa, giving it high resistance to deformation under occlusal load. In contrast, composite resins possess lower mechanical strengths, with compressive strengths typically ranging from 230 to 260 MPa [45] and an elastic modulus between 5.84 and 15.6 GPa. While composite resins offer a degree of resilience, their relatively low fracture toughness - reported to be in the range of 1-2 MPa·m^½^ - can increase the risk of marginal fractures over time [46,47].

Nickel-chromium (Ni-Cr) alloys, commonly employed in metal-ceramic restorations, demonstrate compressive strength values between 450 and 1,000 MPa and possess a high elastic modulus of approximately 210 GPa [47,48]. These properties allow Ni-Cr alloys to withstand substantial functional forces while providing structural support for veneering ceramics.

Gold alloys have long been used in dentistry for their favorable biocompatibility and ease of manipulation. Their compressive strength varies significantly depending on the specific composition and typically ranges from 180 to 1,000 MPa. Furthermore, the modulus of elasticity for gold alloys lies between 90 and 105 GPa, providing a favorable balance between rigidity and flexibility [48].

Lithium disilicate, a glass-ceramic material prized for its aesthetic translucency and strength, has a compressive strength of approximately 300 to 400 MPa and an elastic modulus of almost 95 GPa [49]. These characteristics make it suitable for anterior and posterior restorations where both esthetics and durability are desired.

Table 1 summarises the compressive strength and elastic modulus of the restorative materials used in dentistry, highlighting their mechanical behaviour under functional loads.

**Table 1:** Summary of Compressive Strength and Modulus of Elasticity for Restorative Materials PEEK: polyether ether ketone; Ni-Cr: nickel-chromium Sources: [43,45-49]. Table credits: Created by the authors.

Material	Compressive Strength	Modulus of Elasticity
Composite resins	230-260 MPa	5.84-15.6 GPa
PEEK	250-260 MPa	3-4 GPa
Zirconia	2,000 Mpa	389.5 Gpa
Ni-Cr alloys	>450 MPa to 1,000 MPa	~210 GPa
Gold alloys	180-1,000 MPa	90-105 GPa
Lithium disilicate	300-400 MPa	~95 GPa

Bonding Ability

Effective adhesion to tooth structure is fundamental to the long-term success of restorative procedures. Composite resins achieve micromechanical retention primarily through adhesive systems and typically reach bond strengths in the range of 20-30 MPa [48]. In contrast, ceramic materials require additional surface conditioning, such as hydrofluoric acid etching followed by silanization, to optimize bond strengths and promote durable adhesion to resin-based materials [49]. PEEK, a high-performance polymer, possesses inherently low surface energy. It therefore needs surface modifications like air abrasion or plasma treatment, as well as primer application, to enhance its bondability. Following such treatments, the shear bond strength to composite resin generally ranges between 10 and 20 MPa [50].

Zirconia presents another bonding challenge, as it is resistant to conventional acid etching. To improve its adhesion to resin cements, zirconia typically requires a mechanical surface treatment, such as sandblasting, in combination with chemical priming using phosphate monomers such as 10-methacryloyloxydecyl dihydrogen phosphate (10-MDP). The resulting bond strength usually falls within the range of 15-25 MPa [51]. For Ni-Cr alloys, bonding is primarily achieved via mechanical retention through sandblasting, along with the use of metal primers that promote chemical interaction with the alloy’s oxide layer. Reported bond strengths for Ni-Cr alloys are comparable to those of composite resins and range from approximately 20 to 30 MPa [48]. Similarly, bonding to gold alloys requires specific surface conditioning protocols and using metal primers designed to enhance chemical adhesion to noble metal surfaces [48].

Wear Resistance

The wear resistance of restorative materials is influenced by a complex interplay of mechanical, chemical, and microstructural factors. In composite resins, wear behavior is largely determined by the characteristics of the filler particles, including their size, shape, content, orientation, and distribution. Additionally, the type of monomers used affects the degree of polymerization, which in turn impacts surface hardness. The integrity of the bond between the organic resin matrix and the inorganic fillers is also critical; weak bonding can lead to filler particle detachment and accelerate surface degradation. In combination with the variability in masticatory forces, these factors contribute to the complex wear dynamics observed in composite resins [52].

PEEK, despite having a relatively low elastic modulus and surface hardness, demonstrates wear resistance comparable to that of metal alloys. PEEK has been shown to perform favorably under lateral forces and exhibits an abrasion rate similar to that of resin-based materials when in contact with natural dentition [53]. Zirconia, particularly in its monolithic form, is considered highly wear-resistant. It is less likely to cause excessive wear on the opposing teeth compared to feldspathic porcelain and glass ceramics. Current literature supports the use of a polished surface over a glazed one, as polishing improves long-term wear behavior and reduces antagonistic tooth wear [54].

Ni-Cr alloys are also noted for their high wear resistance, attributable to their inherent hardness and strong mechanical properties [48]. However, proper surface finishing is essential to minimize abrasive wear on opposing dentition while preserving material durability [48-55]. Long valued for their excellent wear resistance, gold alloys have malleability and ductility that minimize their surface degradation over time. Nonetheless, a study by Cha et al. [55] reported that gold alloys exhibited significantly higher wear compared to cobalt-chromium (Co-Cr) alloys, suggesting that alloy selection should be context-specific [55].

Lithium disilicate ceramics offer high wear resistance, although their wear resistance is generally lower than that of zirconia. This difference is attributed to the relatively weaker interfaces within its biphasic microstructure. Interestingly, this characteristic contributes to enhanced machinability that facilitates efficient chairside adjustments and fittings without compromising overall clinical performance [56].

Occlusal design principles

Ideal Occlusal Scheme for Single-Unit Restorations

An ideal occlusal scheme for single-unit restorations is designed to distribute occlusal forces evenly along the long axis of the restored tooth. This alignment helps minimize lateral stresses and reduces the risk of overloading both the restoration and the supporting periodontal structures. One widely accepted approach is the conformative technique, which involves fabricating restorations in harmony with the patient’s existing jaw relationships. In clinical practice, this means constructing the occlusion of the new restoration such that the occlusal contacts of adjacent or opposing teeth remain unaltered [57]. This method is particularly favored for its safety and predictability, as it is less likely to cause complications involving the tooth, the periodontium, the masticatory muscles, or the TMJs [57].

Qureshi and Imran [58] outlined several clinical scenarios where the conformative approach is most appropriate. First, it is suitable when the patient exhibits an existing occlusion that is close to ideal, specifically when a centric occlusion coincides within 1 mm of centric relation, anterior guidance is effectively positioned, and there are no posterior interferences. Second, the approach can be employed even in cases of non-ideal occlusion, provided that the removal of the existing occlusal surface from the tooth being restored does not lead to significant changes in the overall occlusal scheme or altered anterior guidance. Lastly, the conformative method is recommended in the absence of TMDs. In patients with TMD, it should be addressed prior to initiating restorative treatment, as managing TMD may alter the patient's occlusal relationships and require a more comprehensive approach [58].

Cusp-Fossa Relationships and Their Significance

Cusp-fossa relationships describe the spatial interaction between the cusps of mandibular teeth and the fossae of maxillary teeth. This anatomical relationship plays a fundamental role in determining the direction and magnitude of occlusal forces. The multi-cusped nature of posterior teeth enhances masticatory efficiency by increasing the occlusal contact area, which helps distribute occlusal forces more effectively and reduces the risk of stress concentration on specific points [59].

Alterations in occlusal relationships, such as those seen in posterior crossbites, can shift the primary occlusal contacts. In such cases, occlusal forces are transferred from the lingual to the buccal side of the mandibular buccal cusps, resulting in increased apical stress directed toward the buccal aspect [60]. Furthermore, the shape and contour of the occlusal surface significantly affect the absorption of vertical loads. A flat occlusal surface, for instance, is less efficient at dispersing these forces compared to a well-defined cusp-fossa arrangement, leading to greater apical stress and potentially compromising the supporting structures [60].

In a previous study, Wang et al. used photoelastic stress analysis to assess how variations in occlusal surface morphology affect apical stress under vertical bite forces [60]. Their findings indicated that in normal occlusion, apical stress was primarily directed distolingually in posterior teeth, highlighting the role of distal and lingual incline planes of the cusps in bearing the occlusal load. Due to reduced capacity for load dispersion, flattened occlusal surfaces resulted in elevated apical stress. Moreover, well-defined cusp-fossa morphology was shown to more effectively dissipate occlusal loads, thereby minimizing stress concentrations at the apex. Although a minor correlation was observed between the inclination of the tooth’s long axis and the direction of apical stress, occlusal morphology exerted a more dominant influence on stress distribution. Additionally, the study found that the presence or absence of third molars had minimal impact on the overall magnitude of apical stress. However, slight differences were observed in specific regions, such as the second molar area [60].

In essence, maintaining optimal cusp-fossa relationships is essential not only for efficient function but also for the long-term success of restorations. Deviations from ideal occlusal anatomy may result in occlusal interference, increased wear, and potential damage to both natural teeth and prosthetic components [59].

Designing for Functional and Parafunctional Activities

When designing occlusal surfaces for restorations, it is essential to consider both functional activities, such as mastication and speech, and parafunctional habits, including bruxism and clenching. According to Ash [61], restorative planning should proceed under the assumption that patients with bruxism or clenching are likely to continue these behaviors over time [61]. Therefore, restorative designs must incorporate durable occlusal schemes capable of withstanding elevated functional loads, with the goals of minimizing stress on the restoration, natural dentition, and TMJs. Additionally, occlusal adjustments should be directed at eliminating iatrogenic interferences, as these may exacerbate parafunctional habits and contribute to further damage. Occlusal modifications should also be selectively employed as preventive measures to reduce excessive forces on vulnerable teeth, particularly during restorative procedures. The mechanical demands of parafunctional activities, as well as the long-term maintenance of occlusal stability, should be key considerations during treatment planning.

Ash further emphasizes that occlusal therapy should be designed not necessarily to treat the underlying etiology of bruxism, which often remains uncertain, but rather to mitigate its clinical consequences, such as wear and fracture of restorations. Clinical decision-making must therefore integrate evidence-based research with individual patient assessment to ensure that occlusal interventions are tailored effectively to manage associated risks [61]. A thorough diagnostic approach, including clinical evaluation and, when appropriate, sleep laboratory studies, can help assess the severity and impact of bruxism and clenching [62].

To protect restorations and natural teeth from the damaging effects of parafunctional activity, the use of stabilization-type occlusal splints (bite plane splints) is recommended by Lobbezoo and Lavigne [63]. Although their impact on the etiology of bruxism remains inconclusive, these appliances are widely used to control its harmful consequences. Ultimately, the goal of functional occlusion is to direct forces along the tooth’s long axis. If there is a risk of excessive wear, design modifications should be implemented. In general, interocclusal appliances continue to be the most commonly accepted method for preserving both teeth and prosthodontic restorations, despite the current lack of definitive evidence regarding their long-term efficacy [64].

Managing occlusal forces

Occlusal Corrections

Occlusal corrections are essential for functional harmony, improved force distribution, and the long-term success of restorative treatments. Occlusal corrections are indicated in several clinical scenarios. These include improving jaw relationships to establish stable tooth contacts in the intercuspal position (ICP), enhancing individual tooth stability through the smoothing of occlusal contacts, and facilitating lateral and protrusive guidance by modifying plunger cusps, locked cuspal inclines, and interfering contacts. Occlusal corrections are also used to address traumatic occlusion by eliminating interferences that generate excessive loading, thereby reducing the risk of sensitivity, fractures, or tooth mobility. Furthermore, occlusal loads should ideally be directed along the long axes of teeth to minimize lateral forces. These corrections also help promote desirable orthodontic, restorative, or prosthodontic outcomes, either by maintaining existing restorations or by combining occlusal adjustments with selective buildup [65].

Effective occlusal adjustment begins with a comprehensive preclinical phase. This involves verifying the articulation of study casts using transfer records and occlusal indicator wax to identify retruded contact position (RCP) contacts. A dye spacer or highlighting pen is then used to clearly demarcate occlusal contact areas on duplicate casts. Diagnostic occlusal adjustments are performed on the articulated casts to identify the areas suitable for either reduction or addition. The planned adjustments must be minimal and precise, and the selected areas are marked clearly. A thermoplastic vacuum-formed template is fabricated over the unadjusted cast to capture the intended modifications, and it is trimmed to extend approximately 2 mm beyond the gingival margins. The diagnostic phase also helps determine whether additive procedures are required to improve occlusal stability or guidance. Adjustments in RCP should aim for bilateral, evenly distributed stops that do not alter the occlusal vertical dimension. The preclinical adjustments serve as a blueprint that reduces chairside time and increases the accuracy of in-mouth adjustments [65].

The clinical phase begins with intraoral seating of the thermoplastic template to confirm an accurate fit and avoid soft tissue trauma. Areas requiring adjustment will be visible through perforations in the template. Tooth structure is carefully reduced using a sharp scalpel or finishing diamond bur, following the pattern predetermined on the study casts. RCP contacts are refined to achieve well-distributed bilateral stops, and ICP contacts are adjusted to ensure synchronized, balanced occlusal points. Mediotrusive interferences are removed to enable smooth lateral excursions, while laterotrusive and protrusive movements are adjusted to permit free gliding of the maxillary arch. Once adjustments are complete, all treated surfaces are polished, and topical fluoride is applied to promote remineralization and sensitivity reduction. If further restorative procedures are planned, new impressions may be performed to fabricate diagnostic wax-ups based on the modified occlusion [65].

Common failures in single-unit restorations

Single-unit restorations, while generally successful, are subject to a variety of failure modes influenced by mechanical, biological, and procedural factors. According to a study by Cheung [66], an extracoronal restoration is considered a failure if it presents with one or more of the following: technical complications such as restoration or tooth fractures, root fractures, or loss of retention; aesthetic concerns where the patient is dissatisfied with the restoration’s appearance; cariologic issues such as marginal caries; endodontic problems such as periapical involvement or the need for root canal treatment post-cementation; and other causes including tooth extraction for unrelated reasons [66].

A more recent study by Hawthan et al. [67] examined various factors affecting the survival of tooth-supported single crowns, with a focus on biological and technical complications [67]. Their findings revealed that mechanical failures were among the most common, with loss of retention (26.5%) and tooth fractures (12.6%) frequently reported. Other technical complications included framework fractures and periapical destruction. The impact of compromised periodontal and pulpal health was seen in the form of biological issues, such as tooth loss (13.5%), excessive bone loss (4.8%), and the loss of tooth vitality (3.0%). In addition, marginal discrepancies often led to recurrent caries (8.7%), while other issues such as porcelain chipping (3.5%) and esthetic dysfunction (2.6%) were also observed. Notably, several clinical risk factors, such as anterior crown placement, the use of post-and-core restorations in non-vital teeth, and the presence of bruxism, were significantly associated with increased failure rates. These findings underscore the importance of proper tooth preparation, material selection, and occlusal management to maximize the longevity of single-unit crowns.

Inadequate tooth preparation is a well-recognized contributor to restoration failure. When retention and resistance form are insufficient, the likelihood of dislodgement under functional forces increases significantly [68]. Similarly, poor marginal adaptation or suboptimal bonding at the tooth-restoration interface can cause microleakages, creating an environment conducive to secondary caries and eventual failure [69]. Improper distribution of occlusal loads, particularly in the presence of off-axis forces or parafunctional habits like bruxism, can impose excessive stress on restoration margins and cement layers, leading to debonding or material fractures [69]. In addition, a mismatch in mechanical properties between the restorative material and the natural tooth structure may result in stress concentrations that predispose the restoration to fatigue failure over time [70]. Failure to properly adjust occlusal contacts in both static and dynamic movements can cause uneven wear and repetitive overload, and these may ultimately compromise the structural integrity of the restoration [65].

Overall, failures in single-unit restorations are often multifactorial. They typically involve a combination of inadequate preparation, inappropriate material choice, bonding deficiencies, biological complications, and improper occlusal management. A comprehensive and individualized treatment approach is essential to mitigate these risks and ensure long-term clinical success.

Strategies for prevention and management

Preventing failures in single-unit restorations requires a multifaceted and proactive approach throughout all phases of treatment. A thorough pre-treatment evaluation is essential and should assess the remaining tooth structure, pulp vitality, the existing occlusal scheme, and any parafunctional habits such as bruxism [65,66]. Optimal tooth preparation is critical for long-term success; it should provide sufficient retention and resistance while preserving as much coronal structure as possible [68]. Material selection must also be carefully considered. Restorative materials should exhibit favorable mechanical properties and bond strengths appropriate to the functional and biomechanical demands of the tooth being restored [17,18,20].

Precise occlusal adjustments play a vital role in minimizing undue stress. Occlusal contacts should be refined to eliminate interferences and ensure that forces are directed along the tooth’s long axis and evenly distributed across contact points [65,69]. In patients with bruxism or clenching habits, protective interventions, such as the use of stabilization splints or occlusal guards, can help mitigate excessive loading on the restorations [63,64]. Additionally, following accurate bonding and cementation protocols will ensure durable adhesion and a reliable marginal seal, as well as reduce the likelihood of microleakage and secondary caries [48,69].

Patient education is equally important. Clinicians should advise patients on the importance of managing parafunctional habits, maintaining proper oral hygiene, and adhering to follow-up schedules [64]. Routine monitoring of the restoration allows early identification of complications such as loss of retention, fractures, or marginal breakdown. Prompt management of these issues will help ensure the restoration’s long-term survival and functional success [66,67].

## Conclusions

Single-unit restoration failures arise from a combination of mechanical, biological, and procedural factors. Critical contributors include inadequate preparation, inappropriate material selection, bonding issues, and unaddressed occlusal challenges. Identifying and managing patient-specific risk factors, such as bruxism, is essential to long-term success.

Clinicians should emphasize comprehensive pre-treatment assessment, optimal preparation techniques, and materials chosen for their mechanical reliability. Ensuring proper occlusal adjustment and force distribution is equally vital. The incorporation of preventive strategies, including occlusal splints and regular follow-up care, supports the longevity of restorations. Looking ahead, there is a clear need for more robust clinical trials to refine current protocols, clarify the role of individual risk factors, and evaluate the durability of advanced restorative systems.
